# A Novel Method for Real-Time, Continuous, Fluorescence-Based Analysis of Anti-DNA Abzyme Activity in Systemic Lupus

**DOI:** 10.1155/2012/814048

**Published:** 2012-12-05

**Authors:** Michelle F. Cavallo, Anna M. Kats, Ran Chen, James X. Hartmann, Mirjana Pavlovic

**Affiliations:** ^1^Department of Biological Sciences, Florida Atlantic University, Boca Raton, FL 33431, USA; ^2^Department of Biological Sciences, Florida Atlantic University, Davie, FL 33314, USA; ^3^Department of Molecular Genetics and Microbiology, University of Texas at Austin, Austin, TX 78712, USA; ^4^Department of Computer and Electrical Engineering and Computer Science, Florida Atlantic University, Boca Raton, FL 33431, USA

## Abstract

Systemic Lupus Erythematosus (SLE) is an autoimmune disease characterized by the production of antibodies against a variety of self-antigens including nucleic acids. These antibodies are cytotoxic, catalytic (hydrolyzing DNA, RNA, and protein), and nephritogenic. Current methods for investigating catalytic activities of natural abzymes produced by individuals suffering from autoimmunity are mostly discontinuous and often employ hazardous reagents. Here we demonstrate the utility of dual-labeled, fluorogenic DNA hydrolysis probes in highly specific, sensitive, continuous, fluorescence-based measurement of DNA hydrolytic activity of anti-ssDNA abzymes purified from the serum of patients suffering from SLE. An assay for the presence and levels of antibodies exhibiting hydrolytic activity could facilitate disease diagnosis, prediction of flares, monitoring of disease state, and response to therapy. The assay may allow indirect identification of additional targets of anti-DNA antibodies and the discovery of molecules that inhibit their activity. Combined, these approaches may provide new insights into molecular mechanisms of lupus pathogenesis.

## 1. Introduction

Systemic Lupus Erythematosus (SLE) is a chronic, multifactorial, antigen-driven, systemic, autoimmune disease which often presents a broad spectrum of clinical entities. SLE is characterized by the production of an array of inflammation-inducing autoantibodies of IgG and IgM isotypes directed against nuclear antigens, including single-stranded (ss) and double-stranded (ds) DNA. High titers of both antibody classes are involved in disease development and associated with flare [1–4]. Titer of either species allows for differentiation between lupus patients and healthy donors and for monitoring patients in flare and with inactive disease [5–9]. Accordingly, anti-DNA antibody levels in patient sera are used to monitor disease activity and progression [10–12]. However, according to Shoenfeld et al. (1988), although titers vary significantly, anti-DNA antibodies are always detectable in the sera of all healthy mammals [13]. Additionally, methods for quantifying antibody titer can produce greatly varying results from the same serum sample [5] and simply measuring the titer of anti-DNA antibodies does not provide detailed information about antibody functionality or potential pathogenicity. Assaying for antibody hydrolytic activity, in addition to monitoring titer, may allow physicians to better predict changes in disease cycle as well as researchers to illuminate potential roles for abzymes in perpetuation of disease. 

Although sophisticated biosensor-based techniques [14] have been developed for simple detection of the presence of a mixture of both anti-ssDNA and anti-dsDNA antibody levels directly from the blood of SLE patients, assay systems for detecting hydrolytic activity of anti-DNA autoantibodies are still mainly at the electrophoretic level. Most published methods for analyzing the hydrolytic activity of anti-DNA antibody are discontinuous, laborious, and hazardous, often employing radioactivity and/or carcinogenic dyes. For the most part, these assays do not lend themselves to automation nor to study of the reaction kinetics [15–20]. Gololobov et al. [21] have made the only attempt so far at continuous, kinetic analysis of anti-dsDNA antibody hydrolytic activity using the flow linear dichromism technique (FLD). 

In contrast, studies of enzymes that hydrolyze DNA (various DNAases) have provided automated, quantitative measurement of DNA hydrolysis. Modern methods for analyzing DNAse activity range from fluorescence-based (intercalating dyes and fluorescently labeled molecular beacon-based technology) to electrospray ionization mass spectrometry [22–25]. Methods for analyzing autoantibody activity—abzymes in lupus patients—should match the level of sensitivity and ease with which DNAse I, the typical standard control, is analyzed. In order to devise an updated method for measuring abzyme activity, we investigated the utility of a dual-labeled, fluorogenic hydrolysis probe as a substrate for continuous measurement of catalytic activity and compared abzyme activity with a DNAse I control. 

Anti-dsDNA antibodies are found in 70–90% of SLE patients and are considered the hallmark of lupus disease. However, we have chosen to investigate anti-ssDNA antibodies, which present in 30–70% of patients, based on data indicating that they are hydrolytic, nephritogenic, and may serve as strong predictors of flare [3, 4, 26–28]. The significance of anti-ssDNA antibodies in SLE is further supported by data indicating that they are still present after treatment with immunosuppressive therapy which eliminates anti-dsDNA antibodies, investigations in mouse models of nephritogenic lupus in which only anti-ssDNA antibodies were found, and findings that some anti-dsDNA antibodies are not pathogenic [6, 7, 19, 27, 29, 30].

In summary, our basic premises are (1) anti-ssDNA antibodies produced by normal serum donors and those produced by lupus patients can be differentiated based on whether or not they demonstrate hydrolytic activity; (2) these antibodies may be of clinical significance and could prove useful in facilitating diagnosis of lupus disease; (3) it is possible to use hydrolysis probes to determine whether or not purified antibody can cleave DNA. An assay which makes use of hydrolysis probes affords a less hazardous and less laborious method than those currently in use. Additionally, it provides a more sensitive platform in line with the technologies currently available for analysis of DNA hydrolyzing enzymes.

## 2. Materials and Methods

### 2.1. Sample Collection

Sera were separated from fresh, whole blood, drawn intravenously from healthy, normal donors (ND) and Lupus patients (LP) with informed consent and under the guidance of protocols approved by Florida Atlantic University's Institutional Review Board (ref. HO5-278). Serum was harvested by differential centrifugation, aliquoted into 1 mL RNAse/DNAse-free tubes, and stored at −20°C prior to antibody purification. 

### 2.2. Purification of Anti-ssDNA Antibody with Binding Specificity for Poly-(dT) Oligomer

 Natural, polyclonal anti-ssDNA antibodies of the IgG isotype with binding specificity for a purchased oligo-(dT) 20-mer target were isolated using a two-step purification method, previously developed in house [7], which allows for efficient isolation of the intact target molecule. Briefly, the method is based on antibody affinity for runs of thymine (T_5_) and the ability to isolate total IgG with the use of Protein G coated magnetic dynabeads (Dynal Biotech, now part of Invitrogen, Carlsbad, CA, USA). Substantially less laborious than earlier protocols, this method yields antibody of incomparable purity as confirmed at the nanogram level of sensitivity via SDS-PAGE and silver staining. It is also gentle enough to allow further functional analysis of these proteins, which are prone to denaturation under harsh conditions. Purified antibody was stored at −20°C in storage buffer consisting of 25 mM Tris-HCl and 50% glycerol, pH 7.

### 2.3. Confirmation of Antibody Purity

Antibody purity was confirmed as described in Pavlovic et al. [7] by nonreducing SDS-PAGE and silver staining using the Pharmacia PhastSystem with PhastGel Gradient 4–15 separation gels, PhastGel buffer strips, and full-range rainbow molecular weight markers (GE Healthcare, Piscataway, NJ; Owner's manual Separation Technique File no. 130). 

### 2.4. Antibody Quantification

Titers of pure samples were determined as described in Pavlovic et al. [7] by Pierce Micro BCA Assay (Thermo Fisher Scientific/Pierce Chemical Co., Rockford, IL, USA) and a modified ELISA kit developed in house. The ELISA uses ssDNA as substrate in the form of oligo-(dT) 20-mer (Eurofins MWG Operon, Huntsville, AL, USA) bound to Streptavidin microplates (Roche Diagnostics, Indianapolis, IN, USA) with a 4-parameter curve fit model for quantification, which is incorporated into the software of the SpectraMax 190 microplate reader used (Molecular Devices, LLC, Sunnyvale, CA, USA). Serial dilutions of purified human anti-DNA antibody were compared with a human IgG standard. 

### 2.5. Antibody Classification

IgG isotype was confirmed as described in Pavlovic et al. [7] by Western Blot, also using the PhastGel system, with HRP-labeled goat anti-human secondary antibody and TMB substrate (PhastSystem Development Technique File no. 220). 

Presence of IgG_1_, IgG_2_, IgG_3_, and IgG_4_ subclasses was confirmed using a commercial Zymed, human IgG subclass profile ELISA kit (Invitrogen, Carlsbad, CA, USA) [31].

### 2.6. Probe Design

Probe design was based on sequence preference data obtained by Gololobov et al. [15] and X-ray crystallographic data published by Tanner et al. [32]. According to Gololobov's hydrolysis studies in anti-ssDNA mouse monoclonal antibody BV 04-01, anti-ssDNA antibodies prefer C-C regions in ssDNA [15, 27]. Tanner et al. [32] showed that sequence recognition, binding to target DNA, and coordination of the active site occurs at thymine repetitive sequences in their studies of mouse monoclonal anti-ssDNA antibodies. Probe design also accounted for AT rich sequence preferences of DNAse I [15, 27, 32]. The following dual-labeled, fluorogenic oligonucleotide (18-mer) probe (Eurofins MWG Operon, Huntsville, AL, USA) was designed for continuous, fluorescence-based analysis of DNA hydrolytic activity of purified anti-ssDNA antibody with binding specificity for an oligo-(dT) 20-mer: 5′-6-FAM-ATATAGCGC_5_T_5_-DQ1-3′. 

### 2.7. Confirmation of Antibody Hydrolytic Activity

Prior to continuous, fluorescence-based assay, hydrolytic abilities of anti-ssDNA antibodies purified from SLE patient serum were confirmed by Agilent 2100 Lab-on-Chip digital analysis with a DNA7500 microchip (Agilent Technologies, Santa Clara, CA, USA). Hydrolysis of ss herring sperm DNA (obtained by heating and fast cooling) by anti-ssDNA SLE antibody was compared to hydrolysis by a commercial DNAse I positive control (Applied Biosystems, Austin, TX, USA). Parallel DNAse I and anti-ssDNA antibody reactions were analyzed at 37°C within separate reaction buffers consisting of 25 mM Tris-HCl, 50 mM NaCl, 10 mM MgCl_2_, pH 7.5 and 22 mM Tris-HCl, 5 mM MgCl_2_, 0.5 mM CaCl_2_, pH 7.5, respectively, in order to meet requirements of each entity per data published by Odintsova et al. [33]. Enzyme to substrate ratio was 1 : 2 (1 *μ*g of protein = 1 unit of DNAse I) and samples were loaded in volumes of 10 *μ*L.

### 2.8. Confirmation of DNAse I and Abzyme Ability to Hydrolyze Designed Probe Sequence

Cleavage of the oligo 18-mer hydrolysis probe was first analyzed by UV spectrophotometry (A260) to confirm ability of SLE antibodies and DNAse I control to bind and cleave the probe sequence. SLE antibodies were also compared with antibodies purified from normal healthy donors in this experiment. Lupus antibody or DNAse I was combined with probe in optimized enzyme to substrate ratios of 1 : 0.45 and 1 : 2, respectively, within the same distinct reaction buffers specified in Section 2.7. Total reaction volume was 200 *μ*L with a final probe concentration of 1 *μ*g/mL. DNAse trials were conducted at 37°C. UV spectrometry was performed on antibody samples incubated at 4°C, a temperature at which DNAse I is inactive. A total of four lupus patient serum samples and four normal healthy donor serum samples were analyzed in this preliminary confirmation of hydrolytic ability.

### 2.9. Real-Time, Continuous, Fluorescence-Based Hydrolysis Assay

Five trials of the anti-ssDNA lupus antibody, normal healthy donor antibody and control DNAse I reactions were run with samples loaded in triplicate within the same distinct reaction buffers as specified in Section 2.7. Black, flat-bottom, polystyrene, 96 well microtiter plates (MTX Lab Systems, Vienna, VA, USA) were used to prevent cross-contamination. Purchased lyophilized hydrolysis probe was reconstituted in 1X reaction buffer. Hydrolysis probe was combined with DNAse I, LP anti-ssDNA antibody, and ND anti-ssDNA antibody to produce final enzyme to substrate ratios of 1 : 2, 1 : 0.45, and 1 : 0.85, respectively, across samples in total reaction mixture volumes of 200 *μ*L with final probe concentrations of 1 *μ*g/mL. Probe hydrolysis was measured using a Finstruments Fluoroskan II plate reader (MTX Lab Systems, Vienna, VA, USA) with a sensitivity range of pmol-*μ*mol and DeltaSoft3 software. Samples were read top to bottom over an 8 hour assay run at 37°C with fluorescence excitation at 485 nm and emission detection at 538 nm (FAM was read as FITC as directed by Finstruments technical support). Kinetic measurements were carried out every 2 minutes over 30 minutes for DNAse I and every 5 minutes over 8 hours for antibody. Results are reported in absolute fluorescent units. 

### 2.10. Statistical Analysis

Triplicate absolute fluorescent data were averaged at each time step. A one-way analysis of variance (ANOVA) was applied to each data set, DNAse I, and anti-ssDNA antibody, against the control (oligo) to test if there was a significant difference (*α* = 0.05) in the occurrence or intensity of fluorescence, a surrogate for hydrolysis. Time steps (5 min and 1 hour, resp.) were treated as repeated measures. Besides both being compared to the oligo control, the fluorescence of the lupus antibody was also compared to the fluorescence of the normal donor antibody with ANOVA.

## 3. Results

**Figure 1:**
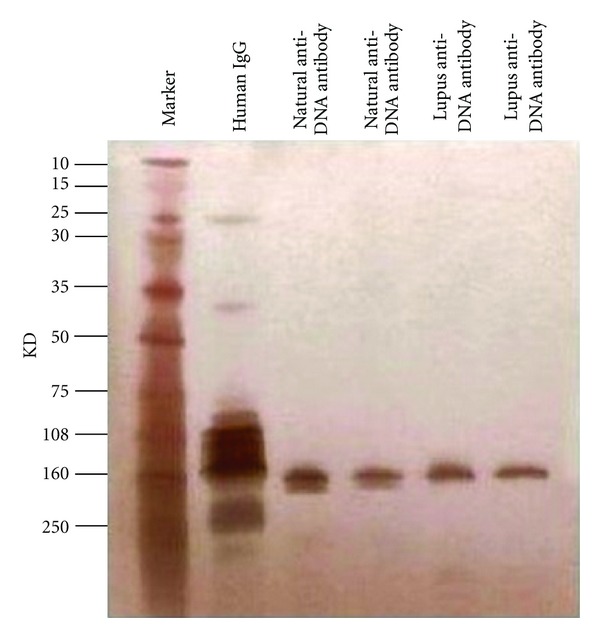
Electrophoretic analysis of the purity of anti-DNA antibody isolated via two-step magnetic bead method. *Purified anti-DNA antibodies from normal healthy serum donors and SLE patients are detected by nonreducing SDS-PAGE and silver staining.

**Figure 2:**
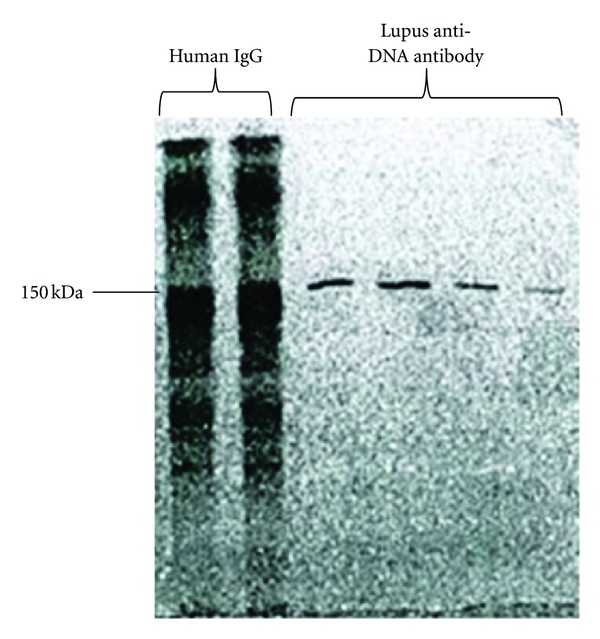
Classification of isolated anti-DNA antibody by western blot. *Western blotting was used to confirm that the proteins isolated using the two-step purification method are IgG.

The purity of the isolated anti-DNA antibodies was determined by nonreducing SDS-PAGE and silver staining. Silver staining with sensitivity to the nanogram level of detection showed an apparent single band with a molecular weight matching IgG in lupus patients and two bands of 150 kDa MW in healthy or normal control serum (Figure 1). Western blot analysis confirmed that the proteins present in the bands are IgG isotype (Figure 2), and subclass profiling by ELISA confirmed the presence of four human IgG subclasses in the purified material [7, 31]. 

**Figure 3:**
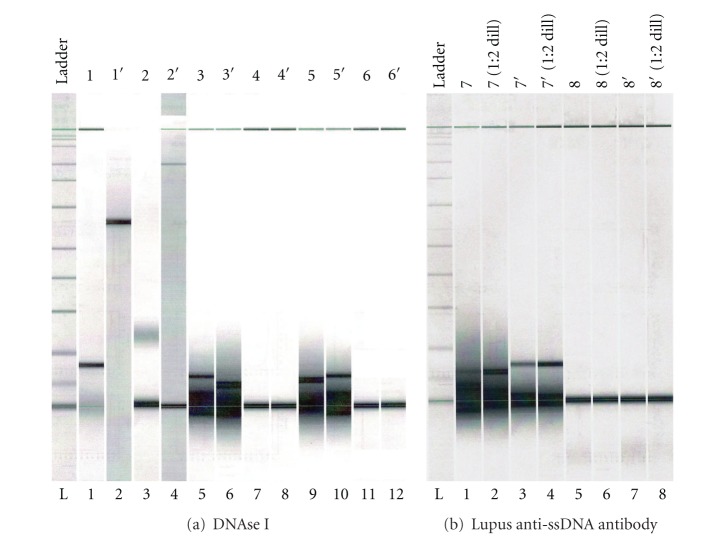
Detection of hydrolysis of ss herring sperm DNA by Agilent 2100 Bioanalyzer (DNA7500 microchip) digital analysis. *Agilent 2100 Lab-on-Chip digital analysis comparison of hydrolysis of ss herring sperm DNA (obtained by heating and fast cooling) by DNAse I control and anti-poly-(dT) ssDNA antibody sample purified from lupus patient serum demonstrates that lupus anti-poly-(dT) ssDNA antibody is hydrolytic. (a) Lane 1: ss herring sperm DNA with DNAse I at T = 0. Lane 2: ss herring sperm DNA, no DNAse I. Lane 3: ss herring sperm DNA with DNAse I at T = 5 min. Lane 4: lambda phage, no DNAse I. Lanes 5, 6: ss herring sperm DNA with DNAse I at T = 30 min. Lane 7: lambda phage with DNAse I at T = 30 min. Lanes 8: lambda phage, no DNAse I. Lanes 9, 10: same as 5, 6. Lanes 11, 12: DNAse I, no substrate. (b) Lanes 1–4: ss herring sperm DNA with lupus anti-ssDNA antibody at T = 3 hrs. Lanes 5–8: lambda phage DNA, no antibody.

Analysis by Agilent 2100 lab-on-chip technology confirmed hydrolytic activity of SLE anti-ssDNA antibody. Results showed hydrolysis of ss herring sperm DNA (500 bp nucleotides) into nucleotides of much smaller MW (25–35–50 bp) by separate addition of both DNAse I and SLE anti-ssDNA antibody to reaction mixtures described in Section 2.7 (Figures 3(a) and 3(b)).

**Figure 4:**
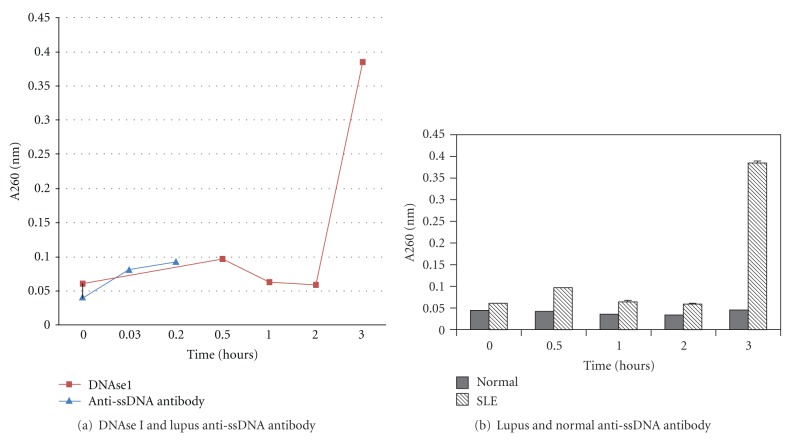
Discontinuous measurement of hydrolysis of oligo 18-mer by DNAse I, lupus patient, and normal donor anti-ssDNA antibody by UV spectrophotometry. *5′-6-FAM-ATATAGCGC_5_T_5_-DQ1-3′ 18-mer hydrolysis probe was incubated with DNAse I or anti-ssDNA antibody purified from lupus patient or normal healthy donor serum. Analysis by UV spectrophotometry demonstrates a change in A260 reading after 3 hours indicating hydrolysis by lupus anti-poly-(dT) ssDNA antibody. Normal healthy donor-derived anti-poly-(dT) ssDNA antibody does not display hydrolytic activity. No change was seen in replicates containing buffer only and those containing substrate only throughout the trials. Endpoint analysis via one-way ANOVA comparing hydrolytic activity revealed a significant difference between activity of antibody purified from lupus patients and normal donors (*F* = 5465.06, *P* < 0.0001). (a) Determination of ability of DNAse I and lupus anti-ssDNA antibody to hydrolyze probe by UV spectrophotometry. The DNAse reaction peaks after approximately 15 minutes and comes to completion in 30 minutes. Activity starts at 0.03 hour, peaks at 0.2 hours, and subsides at 0.5 hours and is much faster than DNA hydrolysis by anti-ssDNA antibody isolated from SLE patient serum. (b) Comparison of ability of purified lupus and normal anti-ssDNA antibodies to hydrolyze probe by UV spectrophotometry. Anti-ssDNA antibodies isolated from normal donors did not demonstrate hydrolytic activity; however, anti-ssDNA antibodies isolated from SLE patients show cleavage ability during the 3 hour incubation time point. These results were consistent across all samples analyzed. *P < 0.01 for normal individuals versus SLE patients.

UV spectrophotometry confirmed the ability of DNAse I and hydrolytic antibody from lupus patients to bind and cleave the designed probe sequence. UV spectrophotometry also demonstrated that anti-poly-(dT) specific ssDNA antibody purified from normal serum did not hydrolyze the probe (Figures 4(a) and 4(b)). This preliminary analysis was performed on four lupus patient serum samples (LP 1–4) and four normal donor serum samples (ND 1–4) and produced results consistent with results of analysis by Agilent and of previous studies which have demonstrated that lupus anti-DNA antibodies hydrolyze DNA while normal anti-DNA antibodies do not [6, 15, 17, 20].

**Figure 5:**
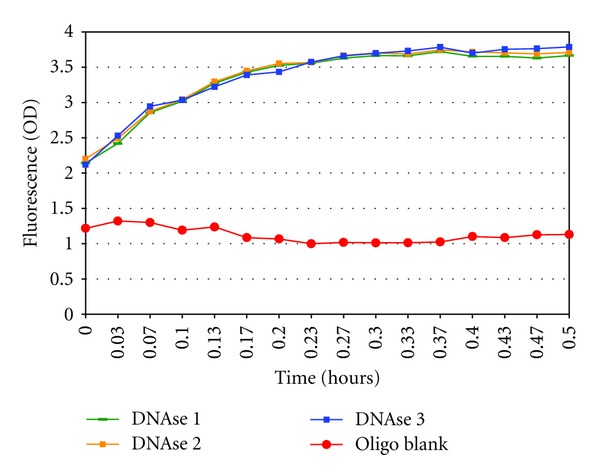
Continuous measurement of oligo 18-mer hydrolysis by DNAse I by fluorescence assay. *Analysis of DNAse I positive control via continuous, fluorescence-based assay on Fluoroskan II (Finstruments) demonstrates that 5′-6-FAM-ATATAGCGC_5_T_5_-DQ1-3′ 18-mer hydrolysis probe is cleaved by DNAse I and that adequate quenching to allow differentiation between cut and uncut probe was achieved. Oligo blank contained all reaction components except for DNAse I (25 mM Tris-HCl, 50 mM NaCl, 10 mM MgCl_2_, pH 7.5, and 1 *μ*g/mL 5′-6-FAM-ATATAGCGC_5_T_5_-DQ1-3′ 18-mer hydrolysis probe). Results were consistent across all trials. DNAse I trials 1, 2, and 3 are displayed as representative trials. Pooled fluorescence data showed statistical significant difference from oligo blank (*F* = 29.78, *P* < 0.0001).

**Figure 6:**
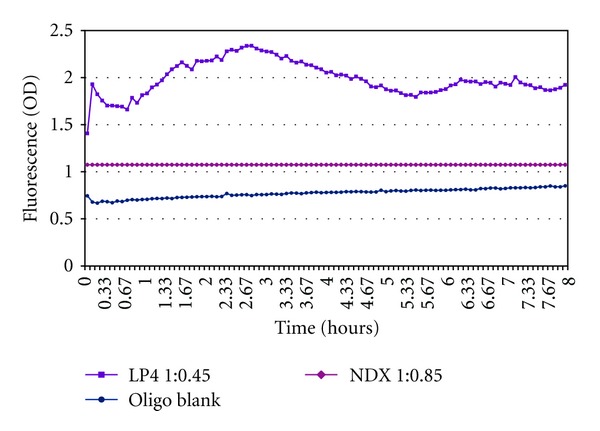
Continuous measurement of oligo 18-mer hydrolysis by lupus patient (LP) versus normal healthy donor (ND) by fluorescence assay. *Comparison of lupus patient-derived anti-poly-(dT) ssDNA antibody (LP4) and normal healthy donor-derived anti-poly-(dT) ssDNA antibody (NDX) via continuous fluorescence-based assay on Fluoroskan II (Finstruments) demonstrates that 5′-6-FAM-ATATAGCGC_5_T_5_-DQ1-3′ 18-mer hydrolysis probe is cleaved by lupus patient-derived antibody but not by normal donor-derived antibody. Oligo blank contained all reaction components except for antibody (25 mM Tris-HCl, 50 mM NaCl, 10 mM MgCl_2_, pH 7.5, and 1 *μ*g/mL 5′-6-FAM-ATATAGCGC_5_T_5_-DQ1-3′ 18-mer hydrolysis probe). ANOVA indicates all three fluorescence patterns of LP4, NDX, and the oligo control are significantly different from each other at *P* < 0.0001.

Hydrolytic activity of DNAse I and purified anti-DNA antibody was next measured by continuous, fluorescence-based hydrolysis assay (Figures 5 and 6). The DNAse I reaction maxima was reached at 15 minutes and the reaction completed at 30 minutes, (Figure 5) while the slower antibody reaction completed in 8 hours (Figure 6). 

Hydrolytic activity was maintained in the antibody storage buffer used, 25 mM Tris, 50% Glycerol, pH 7 at −20°C, and, as was seen in analysis by UV spectrophotometry, both DNAse I and lupus antibody hydrolyzed the probe sequence. A distinct difference between activities of anti-ssDNA antibody purified from lupus patient serum versus normal serum is also detected by varying levels of fluorescence output in continuous, fluorescence-based analysis of hydrolysis (Figure 6). 

Blank buffer controls, which did not contain probe, did not fluoresce. As visualized in Figures 5 and 6, a baseline level of background fluorescence due to incomplete quenching was observed in control samples containing probe without enzyme. Additionally, an increase in fluorescence above the background produced by uncleaved probe is seen in samples containing antibody purified from normal donor serum. However, as the levels of fluorescence in these normal samples remained constant over time, we assume that natural fluorescence of the protein has contributed to this increase in overall fluorescent output. Despite this, samples containing probe hydrolyzed by DNAse I and SLE antibody show a substantial increase in total fluorescence output (mean = 1.93 ± 0.25) above baseline levels produced by probe alone (mean = 0.78 ± 0.04) or by samples containing probe and antibody purified from normal healthy donors (mean = 1.07).

**Figure 7:**
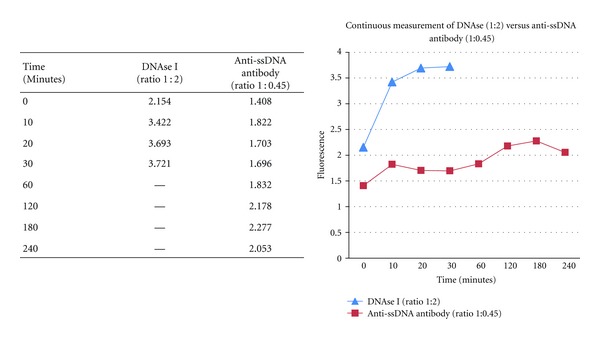
Differences in kinetic behavior of DNAse I and anti-ssDNA lupus antibody in optimized ratios used for detection of ss-DNA hydrolytic activity and expressed in fluorescent units per minutes.

This semiquantitative measurement indicates that DNAse I, at its optimal ratio, displays much faster activity than antibody (reaching a peak of 3.721 units at 30 min. versus 2.277 units at 180 min. for antibody) (Figure 7). Kinetic parameters have not been measured at this time since the main goal of this initial work was to establish the methodology; however, this apparent difference in timing of the reactions and their peaks indicates that they are two different entities. The differences seen between the DNAse I and lupus anti-ssDNA reaction profiles align with differences in reaction mixtures (requirements for coordinators), reaction duration (30 min. versus 8 hrs.), and temperature (4°C versus 37°C) (Table 1).

## 4. Discussion

The use of a dual-labeled, fluorogenic hydrolysis probe as a substrate for measuring anti-DNA antibody hydrolytic activity was inspired by the principles of Fluorescence Resonance Energy Transfer (FRET) and their application in real-time PCR [34]. There are a number of parameters that can affect the assay. Molecular bleaching of the labeled substrate can occur over the long reaction period between antibody and DNA, and this is a disadvantage of the methodology [31]. Despite this, the use of hydrolysis probes was deemed preferable to the use of intercalating dyes which are carcinogenic and could negatively affect the binding affinity for and hydrolysis of DNA by antibody. 

Tanner et al. [32] demonstrated the specific binding of anti-DNA antibodies to thymine polymers via arginine groups using X-ray analysis. The arginine groups are responsible for sequence recognition, and the antibodies bind the DNA at the thymine repetitive sequences (5-mer) via tyrosine side chains within a hydrophobic pocket created by tyrosine and tryptophan within the antibody binding site [15, 27, 30]. While binding affinity of antibody for the probe sequence could have been enhanced due to the presence of the FAM and DQ1 labels, our initial goal was to determine whether presence or absence of hydrolytic capability could be distinguished using this methodology. If binding affinity was enhanced, the labels should have affected both lupus and normal samples; however, we still see a difference in hydrolytic abilities of these entities when comparing their activities against labeled probe. Additionally, since our probe was 18 bases in length (in order to meet requirements for quenching), it would only bind to a single antibody reactive site [19], thus allowing for the monitoring of individual hydrolytic events and targeted analysis of specific sequences. The FLD method, which is the only other continuous method for analyzing DNA hydrolytic antibody activity currently published, requires the use of longer polymers [21]. 

Our lab previously published a novel, two-step method for purification of natural, polyclonal, anti-ssDNA antibodies of the IgG isotype specific for a purchased oligo-(dT) 20-mer target [7]. The method is based on anti-ssDNA antibody affinity for runs of thymine (T_5_) as demonstrated by Tanner et al. [32] and the ability to isolate total IgG with the use of Protein G coated magnetic dynabeads. Streptavidin-coated dynabeads, are incubated with biotynilated oligo-(dT) 20-mer, washed, and then incubated with serum during the first purification step. The beads are washed multiple times prior to elution of bound material. During the second purification step, the eluate is then incubated with protein G beads prior to washing and elution. 

This method yielded highly pure antibody as confirmed at the nanogram level of sensitivity via SDS-PAGE and silver staining and was shown to be of IgG isotype as is demonstrated in our previous publication. We believe it unlikely that a nonantibody protein with binding specificity for the poly-(dT) 20-mer would also have been targeted by protein G and show hydrolytic activity against an alternate sequence previously shown to be a hydrolytic target of anti-ssDNA antibodies [15]. For these reasons, we deemed it unnecessary to incorporate into the purification procedure further treatment to destroy potential contaminating enzymes (e.g., “acid shock” [21]) which could also alter antibody structure and consequently functionality. Our method is gentle, quite rapid, and easy to perform in comparison to previous methods. While the yield is low in comparison to purification methods like those used by Gololobov et al. [21], the yield can be increased by increasing the number of beads used; however, the hydrolysis assay that we have developed can be performed with substantially less-purified antibody than was used in the analysis by FLD method [21]. The levels of potential DNA substrate in our assay ranged from 0 to 1000 ng/mL. This range encompasses that found in blood from healthy patients where DNA and oligonucleotide content is very low (in the range of 10–40 ng/mL), and the range within lupus patients that display levels close to 400 ng/mL [33]. 

The hydrolytic activity of highly purified anti-ssDNA antibodies from lupus patients and the detection of catalytic IgG in other autoimmune diseases, such as Hashimoto's thyroiditis and multiple sclerosis, implicate these antibodies in autoimmune disease pathogenesis [31, 33, 35]. Lupus anti-DNA antibodies demonstrate the ability to penetrate living cells via receptors such as Myosin I, to enter the nucleus, and to trigger cell death in vitro. Hydrolytic anti-DNA antibody could potentially maintain and perpetuate disease by DNA hydrolysis in the nuclei of penetrated cells [36–42]. Future analyses should include cytotoxicity studies and fluorescence-aided tracking of purified, natural hydrolytic antibodies obtained and investigated by the herein described methods.

The methodologies developed within our lab provide a platform for investigation of potential prognostic roles of hydrolytic anti-ssDNA antibodies in flare. Additionally, assaying antibody hydrolytic activity could assist in disease diagnosis and facilitate monitoring of disease state/cycle and responses to therapy. The assay for hydrolysis also allows for indirect identification of pathogenic targets of anti-DNA antibodies and inhibitors of anti-DNA antibody activity. With modifications to the two-step purification procedure and the hydrolysis probe sequence, these analyses can be adapted for investigation of dsDNA antibodies (which show preference for C-G regions [27]), antibody activity against short viral sequences of potential interest in lupus (e.g., Parvovirus B19, HSV, and EBV gene sequences [6, 43]), or any user-defined sequence, as well as comparison of antibody isotypes and subclasses associated with lupus disease. Combined, these methods are less harsh, less hazardous, more rapid, and equally or more specific than existing methods for purification and analysis of DNA hydrolytic antibodies. 

Herein we have demonstrated that hydrolysis probes can be used for nonhazardous, continuous measurement of DNA hydrolytic activity intrinsic to a purified population of anti-ssDNA antibodies produced by SLE patients. We have also shown that, when analyzed by this method, these SLE antibodies are distinguishable from DNAse I based on reaction requirements, speed and duration, and distinguishable from anti-ssDNA antibodies produced by normal donors based on presence versus absence of hydrolytic activity. Further work will include kinetic analyses using the probe methodology and quantification of kinetic parameters as has been done for anti-dsDNA lupus patient antibody by Gololobov et al. [21].

## Figures and Tables

**Table 1:** Summarized comparison of DNAse I (from bovine pancreas, Ambion) and lupus anti-ssDNA antibody hydrolysis reactions.

Parameter/condition	DNAse I	Lupus anti-ssDNA antibody
pH	7.5	7.5
Active temperature range	+22–54°C	+4–37°C
Requirement for Ca^2+^	—	0.5 mM
Requirement for Mg^2+^	10 mM	5 mM
Time to reaction completion	30–40 min	8 hrs

## Abbreviations

Ab: Antibody
6-FAM: 6-carboxyfluorescein
DQ: Dark quencher
ds: Double-stranded
EBV: Epstein-Barr virus
ELISA: Enzyme-linked immunosorbent assay
FRET: Fluorescence resonance energy transfer
HSV: Herpes simplex virus
LP: Lupus patient
ND: Normal donor
ss: Single-stranded
SLE: Systemic lupus erythematosus.
